# Messenger roles of extracellular vesicles during fertilization of gametes, development and implantation: Recent advances

**DOI:** 10.3389/fcell.2022.1079387

**Published:** 2023-01-05

**Authors:** Weisen Fan, Yinghua Qi, Yaqian Wang, Huiting Yan, Xuan Li, Yingjie Zhang

**Affiliations:** ^1^ The First Clinical Medical College of Shandong University of Traditional Chinese Medicine, Jinan, China; ^2^ Affiliated Hospital of Shandong University of Traditional Chinese Medicine, Jinan, China

**Keywords:** extracellular vesicles, embryo, development, implantation, the research progress

## Abstract

Extracellular vesicles (EVs) have become a research hotspot in recent years because they act as messengers between cells in the physiological and pathological processes of the human body. It can be produced by the follicle, prostate, embryo, uterus, and oviduct in the reproductive field and exists in the extracellular environment as follicular fluid, semen, uterine cavity fluid, and oviduct fluid. Because extracellular vesicles are more stable at transmitting information, it allows all cells involved in the physiological processes of embryo formation, development, and implantation to communicate with one another. Extracellular vesicles carried miRNAs and proteins as mail, and when the messenger delivers the mail to the recipient cell, the recipient cell undergoes a series of changes. Current research begins with intercepting and decoding the information carried by extracellular vesicles. This information may help us gain a better understanding of the secrets of reproduction, as well as assist reproductive technology as an emerging marker and treatment.

## 1 Introduction

In 1967, Peter Wolf discovered extracellular vesicles (EVs) (Wolf, 1967). It is a heterogeneous vesicle that is surrounded by lipid bilayers derived from endosomes or cell membranes. Exosomes, Microvesicles, and Apoptotic bodies can be classified based on vesicle diameter, with exosomes (EXOs) measuring 30–150 nm and Microvesicles (MVs) measuring 100 nm-1 μm. Apoptotic bodys (ABs) diameters range from 1 to 5 μm (Merchant et al., 2017). Small EVs have a diameter of less than 100 nm or 200 nm, while large EVs have a diameter greater than 200 nm (Théry et al., 2018). EXOs are the most well-defined EVs, with distinct markers such as CD81, CD89, and other molecules. EXO has a one-of-a-kind biological formation process. To begin, the cell’s plasma membrane shoots inward or outward to form polyvesicles. Vesicles are released after polyvesicles fuse with the plasma membrane to form EVs containing proteins, RNA, DNA, sugars, non-coding RNA, and lipids (Mishra et al., 2021). Through surface-related receptors and specific intercellular adhesion molecules, EVs can specifically recognize target cells. Then, signal transduction occurs *via* endocytosis, fusion, ligand receptor contact, or direct release of vesicle contents, and various target cell functions are regulated (Abels and Breakefield, 2016). Because EV’s structure is stable and resistant to proteases and nucleases in the extracellular environment, it can transmit information from the mother cell to multiple receptor cells throughout the body (Urbanelli et al., 2013). EVs can be produced directly by cells and released into the extracellular environment, or it can enter the extracellular environment *via* transcellular and biological barriers (Fuhrmann, 2020). For example, a large number of endogenous EVs are released from the yolk sac into the peripheral circulation during zebrafish embryogenesis (Verweij et al., 2019). Embryonic EVs can pass through the zona pellucida and enter the culture medium (Vyas et al., 2019). EVs transport also has certain tropism, which may be determined by the receptors it carries and the signals released by the receptor cells (Garofalo et al., 2019; Limongi et al., 2021).

When sperm and egg combine in the oviduct, capacitated sperm pass through the zona pellucida and into the secondary oocyte. Fertilization is completed when the egg pronucleus fuses with the sperm pronucleus. Following that, the fertilized egg develops until it reaches the blastocyst stage, at which point it will be implanted 6–8 days later. Endometrial epithelial cells form large smooth pinopodes in the mid-luteal phase, endometrial stromal cells proliferate and differentiate into decidual cells, and the endometrium enters the “window of implantation” for embryo implantation under the action of estrogen and progesterone. After hatching, the blastocyst enters the endometrium of the “window of implantation” and goes through three stages of positioning, adhesion, and invasion in preparation for implantation (Daikoku et al., 2011). EV’s involvement in vascular biology, immunology, neurology, and reproductive medicine has recently been widely reported. Ovum division, sperm capacitation, embryo development, and implantation all require interaction between the embryo and the mother *via* EVs (Saadeldin et al., 2014; Yáñez-Mó et al., 2015).

## 2 The role of EVs in gametes fertilization

### 2.1 The influence of EVs on oocyte development

Because follicular fluid is the initial environment for oocyte development and oviduct fluid is the final environment for oocyte maturation and fertilization, both follicular fluid and oviduct fluid are important for oocyte development (Da Broi et al., 2018; Saint-Dizier et al., 2019). Oocytes, granulosa cells, and cumulus cells can produce follicular fluid EVs (FF-EVs), which acted as a messenger to transmit information from each cell in the follicle and promote oocyte development (Andrade et al., 2017). When diseases occur in the organism, FF-EVs have a negative impact on oocytes. For example, miRNA-424-5p found in PCOS patients’ FF-EVs can inhibit cumulus cells, inhibit Rb/E2F1 signaling, and is not conducive to oocyte maturation (Yuan D et al., 2021). Of course, the contents of EVs change with menstrual hormone levels on a regular basis. Because the physiological state of follicles, fallopian tubes, and endometrium varies with hormone levels, so do the number of EVs and their contents (de Ávila et al., 2020; da Silveira et al., 2018; Almiñana et al., 2018; Vilella et al., 2015; Yi et al., 2022). The miRNAs contained in the equine FF-EVs differed depending on Deviation, mid-estrous period, and ovulation period, and the majority of these different mirnas were involved in growth and apoptosis pathways (da Silveira et al., 2015). The function of these various mirnas in oocyte development is still unknown. Given what is known, we can only speculate about their role. For example, miRNA-181a expression in FF-EVs is significantly reduced during ovulation in young horses. MiRNA-181 can activate activin receptor IIA, causing SMAD phosphorylation and inhibiting cell proliferation (Zhang et al., 2013). To some extent, the contents of FF-EVs can also reflect reproductive ability. TGF-β was the main targeted pathway of miRNA with differential expression when miRNAs in FF-EVs of young mares and old mares were measured, which was widely involved in cell proliferation, apoptosis, and differentiation (da Silveira et al., 2015; da Silveira et al., 2012). In FF-EVs of aged mares at mid-estrus, the expression of miRNA-23a and miRNA-132 was higher than in young horses, while the expression of miRNA-222 was lower. MiRNA-23a levels rise in patients with premature ovarian failure as FSH levels rise. Furthermore, before follicle maturation, miRNA-132 expression was higher in luteinized follicles than in dominant follicles (da Silveira et al., 2012). miRNA-222 has the ability to regulate the estrogen receptor and Cyclin-dependent kinase 1, as well as promote estrogen secretion (Sang et al., 2013). The connection between miRNA and ovarian function in these FF-EVs are still unknown. Existing studies, however, have revealed a correlation between these miRNAs and ovarian function, albeit without the involvement of EVs.

FF-EVs can regulate oocyte development in the early stages, possibly by regulating the level of cyclic adenosine monophosphate and blocking oocyte meiosis in the foaming stage (Pioltine et al., 2020). When bovine FF-EVs are used to culture bovine cumulus-oocyte complex, it can promote cumulus complex expansion, and larger FF-EVs can also inhibit granulosus cell apoptosis and promote estradiol secretion (Hung et al., 2015; Ying et al., 2023). This could be because miRNA-17b and miRNA-92a in FF-EVs can promote granulosa cell proliferation and cumulus expansion, which is linked to the activation of Src, PI3K/Akt, and MAPK signaling pathways (Hung et al., 2017; Inoue et al., 2020). Oocytes’ *in vitro* maturation rate can be increased when cultured with equine FF-EVs (Gabryś et al., 2022). Furthermore, FF-EVs can mitigate the negative effects of heat shock on oocytes (Rodrigues et al., 2019). In contrast, two studies have shown that the cumulus complex does not expand efficiently when cultured with FF-EVs. As a result, the effect of FF-EVs on the cumulus complex requires further investigation (Matsuno et al., 2017; Rodrigues et al., 2019). The rate of blastocyst formation, implantation, and abortion is determined by the quality of the oocytes. FF-EVs has been shown in studies to increase blastocyst rate while improving oocyte quality (da Silveira et al., 2017; Asaadi et al., 2021). However, the number of studies is small, and more research is needed.

When an egg develops in the oviduct, it is also exposed to oviduct EVs(O-EVs). O-EVs can promote oocyte maturation *in vitro* by upregulating the EGFR/MAPK pathway, reducing reactive oxygen species (ROS) accumulation, inhibiting cell apoptosis, promoting cumulus cell proliferation, and increasing the rate of oocyte maturation (Lee et al., 2020a; b). However, when the concentration of EVs are too high, the incidence of the second meiotic metaphase of egg cells is reduced (Lange-Consiglio et al., 2017). FF-EVs, of course, acts on the oviduct and causes changes in the transcriptome of oviduct epithelial cells. Treatment of oviduct epithelial cells with bovine FF-EVs, for example, increases signaling pathways such as oxidative phosphorylation, thermogenesis, and amino acid biosynthesis, all of which are linked to sperm swimming, fertilization, and embryo growth and development (Hasan et al., 2020). Wang et al. discovered that bovine FF-EVs could promote ovarian cortical stromal cell proliferation, inhibit apoptosis, and increase androstenedione and progesterone synthesis (Ying et al., 2021). MiRNA-31-5p in FF-EVs regulated the follicular growth suppressor gene SFRP4 and promoted ovarian granulosus cell proliferation and progesterone synthesis *via* the WNT/B-CATENIN pathway in another study of FF-EVs in pigs (Yuan C et al., 2021). It is also suggested that EVs acting as messengers may regulate the effects on hormones during oocyte development.

### 2.2 The influence of EVs on sperm fertilization

#### 2.2.1 SP-EVs can both promote and protect sperm fertilization

Seminal plasma EVs (SP-EVs) may improve sperm fertilization ability. Seminal plasma contains EVs produced by the prostate, epididymis, and testis, and healthy EVs have been shown to promote sperm-egg binding. Cat SP-EVs have been shown to improve the fertilization ability and developmental potential of immature spermatozoa, as well as spermatozoa motility after freezing and thawing (Rowlison et al., 2021). SP-EVs have been shown to improve sperm motility after sperm culture, which may be due to the fact that SP-EVs contain many proteins that promote sperm survival and capacitance (Mahdavinezhad et al., 2022). Long-chain fatty acid transport protein 2 (LCFATP2), WAP four-disulfide core Domain protein 8 (WFDC8), and beta-Galactosidase-1-like Protein 4 (GLB1L4) are examples of proteins that can be transferred to sperm. WFDC8 protein, for example, is involved in male reproductive immunity and has anti-microbial properties. The GLB1L4 protein is associated with sperm capacitation, and when the GLB1L4 gene is knocked out, the sperm hyperactivation index and sperm protein phosphorylation level are significantly reduced (Dong et al., 2021; Barrachina et al., 2022). The acrosome reaction is essential for egg fertilization. Many enzymes involved in sperm acrosome reaction are found in SP-EVs, including arachidonic acid 15-lipoxygenase and extracellular diadenosine polyphosphate hydrolase. After fusion with spermatozoa, SP-EVs can stimulate acrosome reaction, making spermatozoa more sensitive to the effect of progesterone (Ronquist, 2012; Dong et al., 2021).

To some extent, SP-EVs can protect spermatozoa. Sperm and seminal plasma are foreign to the female body and be attacked by the reproductive system’s immune system. In the female reproductive system, anti-sperm antibodies recognize not only sperm membrane proteins but also SP-EVs (Allegrucci et al., 2001). Related research found that SP-EVs had an immune-protective effect on sperm. Natural killer cells play an important role in female reproduction. When SP-EVs are co-cultured with natural killer cells, the expression of the natural killer cell receptor 2B4 is reduced, as is the activity of the natural killer cells (Tarazona et al., 2011). Furthermore, high levels of ROS are the primary cause of idiopathic male infertility, and abnormally high levels of ROS are more likely to be found in infertile patients’ sperm samples. Sperm is vulnerable to oxidative stress and can be harmed by high levels of ROS. Currently, it is known that polymorphonuclear neutrophils are the primary producers of sperm ROS, and SP-EVs can reduce sperm ROS production while increasing sperm antioxidant capacity, which may be due to SP-EVs hardening the membrane of polymorphonuclear neutrophils. Glutathione S-transferase 2 regulation reduces the production of intrinsic ROS (Saez et al., 1998).

#### 2.2.2 EVs found in the normal female reproductive system can aid in sperm fertilization

The semen transports the sperm into the woman’s vagina after it has been produced. The sperm then travels through the uterus, enters the fallopian tube, and combines with the egg cell to complete fertilization (Saint-Dizier et al., 2020). The environment in the uterus and fallopian tube is critical for sperm survival, transport, and capacitation. This decisive effect is most likely carried out *via* the oviduct and the EVs present in the uterus. Cat O-EVs can bind to spermatozoa at the acrosome and middle part, increasing sperm motility and maintaining acrosome integrity, as well as improving sperm fertilization ability. This could be due to the many proteins found in O-EVs that promote sperm motility and fertilization, such as oviduct-specific glycoprotein, T-complex protein 1 subunit gamma, and T-complex protein 1 subunit alpha (Ferraz et al., 2019). Plasma membrane calcium atpase 4(PMCA4) is a membrane protein that serves as the primary calcium ion discharge pump in sperm. When PMCA4 is missing, it causes severe hyperactivity and loss of sperm movement, which leads to infertility. Mice O-EVs can be absorbed by spermatozoa, transferring PMCA4 to them, promoting calcium outflow, and inducing sperm capacitation (Al-Dossary et al., 2013). Notably, almost all PMCA4a was found in uterine and oviduct fluid EVs.The content of PMCA4a increases by about threefold when cultured with EVs from uterine and oviduct fluid (Al-Dossary et al., 2013). The mechanism by which EVs transfer PMCA4 and other transmembrane proteins to the sperm plasma membrane may be related to the pro-integrinαVβ3 and Integrinα5β1 effects (Al-Dossary et al., 2015). When PMCA4 is absent in sperm, mice O-EVs can compensate by up-regulating Plasma membrane calcium atpase 1 (PMCA1), resulting in calcium ion efflux (Okunade et al., 2004). MiRNA-34c-5p can be transferred to the sperm head by O-EVs. Because the first cleavage of the zygote requires miRNA-34c-5p acting on the centromere, and miRNA-34c-5p can only be obtained from sperm, O-EVs can indirectly affect zygote development *via* sperm (Fereshteh et al., 2018).

Furthermore, intrauterine EVs(I-EVs)can deliver Hyaluronidase PH-20 (SPAM1) to the sperm membrane, and the uptake of SPAM1 by sperm *in vitro* is a sign of sperm maturation. SPAM1 protein can improve spermatozoa binding to hyaluronic acid, increase oocyte cumulus penetration, and induce acrosome reaction related to calcium signal to promote fertilization (Griffiths et al., 2008a). In comparison to the secreting stage, EVs secreted by endometrial cells in the proliferating stage can improve sperm capacitation and fertilization ability, which may be related to EV-induced protein tyrosine phosphorylation of sperm (Murdica et al., 2020). After entering the female reproductive system, sperm will first come into contact with the vaginal environment, and vaginal EVs (V-EVs) also has an effect on sperm. V-EVs contain proteins that inhibit premature sperm capacitation and acrosome reaction, which can increase the likelihood of progesterone-induced sperm acrosome reaction (Fereshteh et al., 2019). V-EVs also contains PMCA4a, which sperm can use (Al-Dossary et al., 2013). The exciting part is that eggs may be able to communicate with sperm *via* EVs before fertilization. According to Miyado et al.(2008) mature eggs can still deliver EVs to sperm. When spermatozoa penetrate the cumulus, they fuse, and this step is aided by the ovum’s secretion of EVs. Furthermore, after follicular fluid enters the fallopian tube following ovulation, FF-EVs may promote sperm motility and fertilization (Sysoeva et al., 2021). Surprisingly, oocytes and cumulus cells can take up SP-EVs, and rabbit SP-EVs can promote cumulus expansion and affect mRNA expression of cumulus cells and oocytes, including PTGS2 and NTRK, which are involved in oocyte development and maturation (Abumaghaid et al., 2022). SP-EVs can also be taken up by porcine cumulus cells, but not by oocytes. In cumulus cells, these EVs can regulate the expression of HAS2 and steroidogenesis associated with oocyte maturation, as well as CYP11A1 and HSD3B1 (Mateo-Otero et al., 2022). This raises the question of whether SP-EV plays a role in oocyte maturation after male and female life.

## 3 The role of EVs in embryonic development

### 3.1 Normal O-EVs may promote embryonic development

The sperm and egg combine to form a zygote, which develops into an early embryo over time. The oviduct is the early embryo’s growth and development environment, and oviduct fluid is an important medium of communication between the oviduct and the embryo (Hasan et al., 2020). O-EVs are an important messenger in this communication process because EVs can cross the zona pellucida. For example (Laezer et al., 2020), the volume of O-EVs change with hormonal levels during the menstrual cycle, and the volume of porcine O-EVs are smaller in the late ovulatory period than in the late oestrus and late oestrus. The protein content of O-EVs change over time. When compared to estrus, syntaxin-2 and syntaxin-binding protein 1 are upregulated during ovulation. These two proteins are involved in sperm acrosome reaction and sperm-oocyte membrane fusion. Embryo implantation alters the types and contents of RNA, protein, and miRNA in O-EVs. When the differences in miRNA expression in oviduct EVs between pregnant and non-pregnant cows were compared, it was discovered that eight miRNAs were down-regulated in pregnant cows, including miRNA-126-5p, miRNA-129, and miRNA-345-3p, while miRNA-331-5p was up-regulated in non-pregnant cows. PI3K/AKT, mTOR, and MAPK are among the signaling pathways regulated by these differentially expressed miRNAs (Mazzarella et al., 2021). The PI3K/AKT signal transduction pathway, for example, can regulate the growth and survival of embryonic cells prior to implantation, and the PI3K-Akt subunit in the pathway is expressed from the zygote to the blastocyst stage and is involved in blastomere proliferation (Fiorenza et al., 2020).

EVs produced by healthy fallopian tubes are beneficial to embryo development. In pigs, for example, O-EVs can promote embryonic development by increasing blastocyst formation rate and attachment ability (Fang et al., 2022a). The contents of O-EVs may be the cause of the positive effect, not only promoting early embryo development but also alleviating the embryo’s damage from adverse factors. ROS can harm cells, impair embryo development, and reduce embryo transfer success rates (Tsukazawa et al., 2021). 5-methylcytosine (5-MC) is a well-studied DNA modification base in prokaryotes and eukaryotes that is involved in nuclear reprogramming, embryonic development, and gene expression regulation (Kumar et al., 2018). When O-EVs are used to culture embryos, the blastocyst rate and hatching rate of embryos are significantly increased, the levels of ROS and 5-MC are decreased, the inner cell mass (ICM)/trophoblast cell (TE) index is increased, and the apoptosis rate is decreased, which may be related to the melatonin contained in O-EVs (Qu et al., 2020). During Apoptosis in mouse embryos cultured with O-EVs, Apoptosis Regulator Bcl-2 (Bcl-2) expression was increased while Apoptosis Regulator BAX (BAX) expression was decreased, which also promoted embryo differentiation and increased the birth rate after embryo transfer (Qu et al., 2019). Porcine O-EVs can decrease caspase-3 gene expression, decrease blastocyst cell apoptosis, and increase blastocyst formation rate. This could be caused by lowering the expression of the sXBP1, ATF4, and GRP78 genes in order to reduce embryonic ER stress (Fu et al., 2022). Furthermore, O-EVs have an energy regulation effect on embryo development. The addition of O-EVs to embryo culture medium has been shown to inhibit Pyruvate dehydrogenase kinase-mediated expression of isoform 4 and Sirtuin 4. Up-regulation of Pyruvate dehydrogenase and Glutamate dehydrogenase expression can increase Pyruvate flux in the tricarboxylic acid cycle, dynamically regulate embryo mitochondrial metabolism, and improve blastocyst quality and hatchability (Sidrat et al., 2020).

### 3.2 Normal I-EVs can promote embryonic development

The intrauterine fluid contains various enzymes, hormones, glucose, and amino acids required for embryonic development, and it serves as a conduit for communication between the mother and the embryo, determining the embryo’s development and survival (Silva et al., 2021). I-EVs are crucial in embryo implantation. When Lv et al. (2018). Cultured embryos with pregnant mouse uterus flushing solution, they discovered that the pregnant group’s EVs content were higher than the non-pregnant group’s. Furthermore, the I-EVs of pregnancy group had higher levels of miRNA-21 expression, which can promote embryonic development. The final culture results showed that I-EVs during pregnancy could increase the ICM/TE index, decrease BAX expression in embryos, and increase Bcl-2 and Octamer-binding protein 4 expression (OCT4). OCT4 controls gene expression in the early stages of embryo formation and plays a critical role in ensuring the number of pluripotent stem cells as a key protein to maintain the pluripotency of embryonic stem cells (Stirparo et al., 2021). However, the effects of EVs produced by the uterus in different states on embryo development vary. When EVs of endometritis cows were compared to that of healthy cows, 52 miRNAs were down-regulated and 66 miRNAs were up-regulated in the EVs of endometritis group, and I-EVs of endometritis could reduce embryo hatching rate (Wang et al., 2019). Similarly, when I-EVs from recurrent implantation failure (RIF) patients are used to incubate embryos, the total number of embryonic cells, hatching rate, and invasiveness are reduced when compared to I-EVs from healthy women (Liu et al., 2020a). Thus, the contents of EVs may play an important role in embryonic development.

EVs secreted by embryos may have an effect on embryo development in addition to the presence of EVs that can affect embryo development. Relevant research has shown that the presence of miRNA-378a-3p in EVs secreted from the blastocyst stage can increase the number of embryonic trophoblast cells and inner cells, as well as improve the hatching rate (Pavani et al., 2022). Surprisingly, SP-EVs may influence early embryo development. This does not necessarily imply that SP-EVs have the same effect in humans. The addition of SP-EVs to a medium containing mouse oocytes and sperm, on the other hand, can significantly increase the rate of blastocyst formation, reduce cell apoptosis, and increase ICM/TE in embryos (Ma et al., 2022). These findings also suggest that various seminal plasma components may have beneficial effects on embryo development and later embryo development when cultured *in vitro*.

## 4 The role of EVs in embryo implantation

### 4.1 The EVs are responsible for communicating between the embryo and the uterus

#### 4.1.1 Luteal phase I-EVs can aid in embryo implantation

Because embryo implantation failure accounts for 75% of pregnancy failure in humans, embryo implantation is a critical step in achieving a successful pregnancy (Pavani et al., 2022). The requirements for embryo adhesion in this process are relatively stringent, requiring both sufficient implantation ability of the embryo and endometrium receptivity. Endometrium receptivity, in particular, requires an appropriate level of inflammation, which first ensures that the embryo is protected from the maternal immune system before implantation, and then requires a maternal endometrial inflammatory response after implantation to promote embryo invasion (Franasiak et al., 2021; Sehring et al., 2022). However, the embryo’s implantation ability and the endometrium’s receptivity must be obtained concurrently, which is more difficult. I-EVs can transmit embryo and endometrium information and is a messenger of signal communication between mother and embryo, which is critical in the embryo implantation process.

During pregnancy, I-EVs primarily consist of EVs produced by the endometrium and EVs produced by the embryo, but it is primarily produced by uterine gland epithelial cells (O'Neil et al., 2020; Hu et al., 2022). The I-EVs also varied with the mother’s estrous cycles, and the embryo can respond differently to endometrial EVs (EM-EVs) at different times to prepare for implantation. EVs secreted by the 13th estrous cycle in the sheep uterus can increase IFNT production of trophoectoderm cells and promote trophoectoderm cell proliferation (Ruiz-González et al., 2015). I-EVs at estrous cycle or day 9 of pregnancy can promote embryonic trophoblast cell proliferation and migration, whereas I-EVs at day 12 or 15 of pregnancy can inhibit embryonic trophoblast cell migration and proliferation (Hu et al., 2022). To simulate the endometrium in the luteal phase, treating human endometrium epithelial cells with estrogen and progesterone to produce EVs can increase trophoblast adhesion and invasion ability, which may be related to EM-EVs upregulating cell-cell adhesion and the cadherin-binding function in trophoblast cell spheroids (Evans et al., 2019). In this process, the “cargo” carried by the EVs can regulate the embryo. For example, miRNA-100-5p in EM-EVs activates FAK and C-jun N-terminal kinase (JNK), promoting trophoblast migration and invasion as well as angiogenesis (Tan et al., 2020a).

The different effects of I-EVs on embryos at different stages may be due to different hormone levels acting on the “cargo” carried by EM-EVs at different stages. Progesterone can not only increase the number of EVs produced by the endometrium, but it can also regulate the miRNA content of EVs. Progesterone-induced miRNA may promote embryo adhesion by regulating PI3K/AKT, BMP, and small RNA post-transcriptional silencing (Burns et al., 2018). Endometrial epithelial cells cultured with estrogen and progesterone produced EVs with higher levels of Laminin subunit alpha-5 (LAMA5) than endometrial epithelial cells cultured with estrogen alone. During embryonic development, LAMA5 mediates cell attachment, migration, and tissue entry. Endometrial epithelial cells cultured with estrogen and progesterone can enhance embryonic trophoblast cell adhesion and up-regulate the expression of FAK and FAK-TYR397. This could be mediated by EVs’ “command” *via* activation of the active adhesive FAK signal (Greening et al., 2016). Similarly, progesterone may affect the contents of EVs produced by embryos (EO-EVs). The EVs produced by trophoblast cells cultured with progesterone is more effective than that produced by trophoblast cells cultured without progesterone in improving IntegrinαV, Integrinβ3, and Protein WnT-7α and decreasing MUCin-1 of uterine epithelial cells (Su et al., 2022).

#### 4.1.2 EVs produced by an abnormal uterus are not conducive to embryo implantation

I-EVs will not be conducive to embryo implantation if the uterus is abnormal. When I-EVs from RIF women were used for mouse embryo culture, but also weakened their invasion ability (Liu et al., 2020a). This could be because EM-EVs in RIF patients reduces trophoblast cell proliferation, migration, and invasion, resulting in implantation failure (Liu et al., 2021). Furthermore, abnormal cytokine and leukocyte expression in endometritis may impair the endometrium’s immune tolerance to the embryo, which is not conducive to the invasion of the embryo’s survival trophoblast, leading to infertility, Recurrent spontaneous abortion (RSA) and RIF (Buzzaccarini et al., 2020; Franasiak et al., 2021). The use of lipopolysaccharide to interfere with endometrial epithelium as a model for endometrial inflammation. EVs produced by it can inhibit the migration of bovine embryonic trophoblast cells and suppress the expression of CDX2, ER, and NANOG genes involved in bovine embryonic development (Wang et al., 2021a). Uterine fluid from a successful pregnancy benefits the embryo. ITGA5, CXCL10, and CXCL11 expression can be significantly up-regulated when cultured with I-EVs from successfully pregnant pigs, whereas ID2 expression can be significantly down-regulated. ITGA5 is a key gene in embryo implantation, and its high expression indicates endometrial receptivity (Ahmad et al., 2018). CXCL10 plays a role in the recruitment of immune cells to the endometrium during embryo implantation as well as the establishment of an immune tolerance environment in the embryo (Złotkowska and Andronowska, 2019). CXCL11 can cause trophoblast cells to migrate, endometrial stromal cells to proliferate, and endometrial epithelial cells to die (Hirota et al., 2006). ID2 is a transcription factor inhibitor that regulates cell differentiation, proliferation, and apoptosis (Xie et al., 2010).

### 4.2 EMT is closely related to the information carried by EVs

The epithelial-mesenchymal transition (EMT) is critical for embryo implantation. Through EMT, the trophoblast gains adhesion and invasion ability, whereas the epithelial-mesenchymal transition of endometrial epithelial cells causes the cell to lose polarity, recombines adhesion molecules on the cell membrane, and promotes endometrium-embryo adhesion (Uchida et al., 2007). As a result, an abnormal EMT between endometrial epithelial cells and trophoblast cells can cause embryo implantation failure (Gou et al., 2019). Communication between the embryo and the endometrium is required prior to implantation for synchronization. EVs are particularly important in the process of EMT. EVs produced by placental trophblast cells can also carry miRNA-1290, which can regulate the LHX6 gene in endometrial epithelial cells and suppress E-cadherin expression, promoting endometrial EMT (Shi et al., 2021). During embryo implantation, aldehyde reductase (AKR1B1) and macrophage-capping protein (CAPG) were up-regulated in the embryos, which also existed in EO-EVs. Both AKR1B1 and CAPG have the ability to promote the EMT process (Nakamura et al., 2016). EMT is the process by which an embryo invades the endometrium and is detected by maternal autoimmunity. Surprisingly, the number of M1 macrophages was increased in the decidua of RSA patients. However, EVs released by M1 macrophages can inhibit trophoblast EMT by promoting E-cadherin expression while inhibiting N-cadherin and vimentin expression (Ding et al., 2021). This process could be linked to TRAF6 downregulation by EVs containing miRNA-146a-5p and miRNA-146b-5p. In normal pregnancy, EVs released by decidual stromal cells increase the invasiveness of trophoblast cells after being ingested by trophoblast cells. This could be because EVs in decidual stromal cells promote EMT by upregulating the expression of phosphorylated Smad2, phosphorylated Smad3, and N-cadherin (Liu et al., 2020b).

### 4.3 EVs regulate the inflammatory and immune environments of embryo implantation

The inflammatory response of the endometrium prior to implantation is required for implantation. EVs produced by embryonic trophoblast cells recruit monocytes in a dose-dependent manner, increasing monocyte secretion of IL-1β, IL-6, and TNF-α to create an inflammatory environment in the uterus prior to pregnancy (Atay et al., 2011). Furthermore, EVs produced by placental trophoblast cells can significantly increase TNF-α, IL-6, and IL-8 expression in endometrial epithelial cells, resulting in an inflammatory uterine environment and increased angiogenesis to promote embryo implantation (Shi et al., 2021). Furthermore, mild endometrial inflammation may provide embryo protection. Mesenchymal stem cells are well known for their ability to regulate the immune microenvironment and influence the function of immune effector cells (Song et al., 2020). However, inflammation and implantation signals induce mesenchymal stem cells in peripheral blood under normal conditions (Calle et al., 2021a). Without recruiting embryonic stem cells, EVs secreted by embryonic trophoblast cells can recruit mesenchymal stem cells in the endometrium and peripheral blood. As a result, stem cells recruited by inflammatory factors produced by trophoblast EVs and trophoblast EVs can inhibit T lymphocyte proliferation, reducing maternal immune rejection of the embryo (Calle et al., 2021b). Despite the fact that embryo implantation necessitates an inflammatory response in the maternal endometrium, more does not appear to be better. For example, the previously mentioned endometrial inflammation is not conducive to embryo implantation. Other research has discovered that M1-M, which has an inflammatory promoting function, is significantly increased in the decidua tissue of RSA patients (Zhu et al., 2020). Furthermore, miRNA-196a-5p was found to be highly expressed in EVs produced by trophoblast cells in RSA patients’ villus tissue. EVs can transfer miRNA-196a-5p into macrophages, and the NF-κB pathway induces macrophage M1 polarization and secretes TNF-α to inhibit trophoblast development (Zhang et al., 2022). As a result, appropriate endometrial inflammation is critical to implantation success.

**FIGURE 1:**
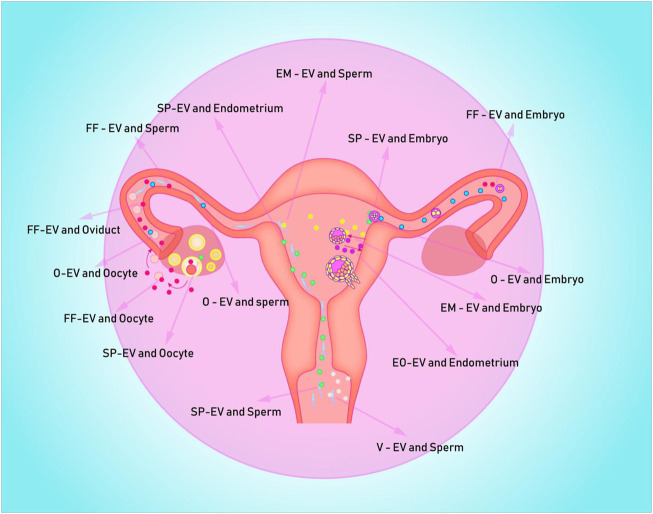
Depicts potential EVs messenger pathways in the human uterus for embryo formation, development, and implantation. EV is represented by the colored spheres. **(A)** The blue ball is the O-EVs, which transports information from the oviduct to the oocyte, sperm, and early embryo; **(B)** The red sphere represents FF-EVs, which can communicate information from the follicle to the fallopian tube, sperm, and early embryo; **(C)** The white ball represents the V-EVs, which can transmit vaginal information to the sperm; **(D)** The green sphere represents the SP-EVs, which is capable of transmitting seminal plasma information to sperm, endometrium, and follicle; **(E)** The purple spheres are mostly EO-EVs, which can send data from the embryo to the maternal endometrium; **(F)** The yellow spheres represent EM-EVs, which transmit information from the endometrium to sperm and embryos.

Seminal plasma not only protects sperm from maternal immune damage, but it also makes embryo implantation easier. This could be accomplished by inducing inflammation in order to improve endometrium receptivity and promote embryo implantation in the uterus. SP-EVs, for example, can promote decidualization of endometrial stromal cells *in vitro*, which may be due to the activation of the IL-11 signaling pathway by SP-EVs proteins (George et al., 2020). Although the mechanism of inflammation is unknown, endometrial epithelial cells treated with SP-EVs have significantly activated inflammatory and immune signaling pathways (Bai et al., 2018). It is also worth noting that the occurrence of inflammation may be related to endometrial stromal cell secretion of inflammatory factors or the transformation of macrophages to M1-M caused by EVs in seminal plasma (Paktinat et al., 2019; Paktinat et al., 2021). Seminal plasma, on the other hand, causes immune rejection or tolerance in the maternal endometrium, which is closely related to the balance of immune-related cells such as dendritic cells, macrophages, and neutrophils (Schjenken et al., 2015). Mouse SP-EVs can inhibit dendritic cell maturation in endometrial tissues, which may increase embryo implantation rates by establishing immune tolerance (Wang et al., 2021b). It should be noted that the extent to which SP-EVs inhibited maternal dendritic cell maturation varies with age. SP-EVs from old mice have weaker inhibition of dendritic cells in the endometrium, higher levels of IL-6, TNF-α, and IL-1β in the uterine cavity, lower levels of IL-10 and TGF-β, and a lower implantation rate of embryos (Wang et al., 2021b). This suggests that the pro-embryo transfer effect of SP-EVs may vary with age. Figure 1; Table 1 depicts a detailed description of the possible messenger roles of EVs at various stages of embryo formation, development, and implantation.

**TABLE 1:** EV’s potential role as a messenger in the human uterus during gametophyte fertilization, early embryonic development, and implantation.

Reproductive process		Results	Source of EVs	References
	Egg cell	Bovine FF-EVs can regulate the arrest of oocyte meiosis, which may be related to the contents of FF-EVs that can regulate cAMP levels	FF-EV	Pioltine et al. (2020)
		Bovine FF-EVs promoted cumulus oocyte complex expansion and increased the expression of PTGS2, PTX3, and TNFAISP6 in both bovine and mouse cumulus oocytes		Hung et al. (2015)
		Porcine FF-EVs contain miRNA-17b and miRNA-92a, which may promote oocyte development		Inoue et al. (2020)
		Bovine FF-EVs can be taken up preferentially by granulosa cells, promoting their proliferation. This could be linked to SRC, PI3K-Akt, and MAPK signaling		Hung et al. (2017)
		Horse cumulus cells and oocytes can internalize equine FF-EVs, promoting oocyte maturation		Gabryś et al. (2022)
		Heat shock can be mitigated by bovine FF-EVs on cleavage and blastocyst development		Rodrigues et al. (2019)
		In pigs and mice, porcine FF-EVs had no effect on cumulus expansion		Matsuno et al. (2017)
		Bovine FF-EVs not only promote oocyte maturation, but also increase the expression of miRNA-631 in embryos, cause changes in the overall DNA methylation and hydroxymethylation levels of embryos, and promote blastocyst formation		da Silveira et al. (2017)
		Bovine FF-EVs have the ability to change the transcriptome of fallopian tube epithelial cells, and some of these functional pathways are linked to sperm survival, fertilization, and early embryonic development		Hasan et al. (2020)
		Bovine FF-EVs can stimulate ovarian cortical stromal cell proliferation as well as the synthesis of androstenedione and progesterone		Ying et al. (2021)
		Bovine FF-EVs can inhibit granulosa cell apoptosis while increasing estradiol secretion		Ying et al. (2023)
		Through the WNT/B-CATENIN pathway, miRNA-31-5p found in porcine FF-EVs can regulate the follicle growth inhibitory gene SFRP4 and promote the proliferation and progesterone synthesis of ovarian granulosa cells		Yuan D et al. (2021)
		miRNA-424-5p in PCOS patients’ FF-EVs can block the Rb/E2F1 signaling pathway and inhibit granulosa cell growth		Yuan C et al. (2021)
		MiRNA types and expression levels differ in FF-EVs of horses at various estrus stages, and some of the action pathways of miRNAs with differential expression are associated with oocyte maturation		da Silveira et al. (2015)
		MiRNAs differ in the FF-EVs of young and old horses, and part of the action pathways of miRNAs with differences are related to oocyte maturation		da Silveira et al. (2012)
		Bovine FF-EVs and O-EVs can improve embryo blastocyst quality and number of embryonic cells while inhibiting cell apoptosis		Asaadi et al. (2021)
		The content of FF-EVs can be used to assess the quality of oocytes		Rodrigues et al. (2019), Battaglia et al. (2020), da Silveira et al. (2021), Gad et al. (2022), Zhang et al. (2021), Martinez et al. (2018)
		Through the EGFR/MAPK pathway, dog O-EVs may promote oocyte development, cumulus cell proliferation, and cumulus oocyte complex expansion	O-EV	Lee et al. (2020a)
		Dog O-EVs can promote oocyte maturation, but at high concentrations, it inhibits oocyte maturation		Lange-Consiglio et al. (2017)
		Bovine O-EVs may inhibit the expression of CASP3 and p-NFκB, which are involved in cumulus cell apoptosis, and promote the expression of CX43, CX37, HAS2, PTX3, and GREM1, which are involved in COC expansion		Wei et al. (2022)
	Sperm	SP-EVs can accelerate immature sperm fertilization, improve early embryo formation after sperm fertilization, and improve sperm motility after freezing and thawing	SP-EV	Rowlison et al. (2021)
		SP-EVs can improve sperm motility, morphology, and viability after cryopreservation, as well as increase antioxidant capacity and reduce DNA damage		Mahdavinezhad et al. (2022)
		SP-EVs can transport epididymid-derived proteins for sperm, which can regulate the sperm proteome and participate in sperm maturation		Barrachina et al. (2022)
		SP-EVs have the ability to deliver progesterone receptors, cyclic adenosine diphosphate-ribose receptors, ryanodine receptors, and other Ca2+ signaling tools to sperm, stimulating acrosome reaction and progesterone sensitivity		Park et al. (2011)
		SP-EVs are recognized by the female immune system		Allegrucci et al. (2001)
		SP-EVs may protect sperm from immune attack by reducing NK cells activity in the female reproductive system		Tarazona et al. (2011)
		SP-EVs may boost sperm antioxidant capacity by acting on polymorphonuclear neutrophils		Saez et al. (1998)
		Cumulus expansion can be aided by rabbit SP-EVs		Abumaghaid et al. (2022)
		Cumulus cells can take up porcine SP-EVs, but oocytes cannot		Mateo-Otero et al. (2022)
		SP-EVs can promote sperm capacitation and the production of endogenous ROS.		Wang D et al. (2022)
		SP-EVs can assess sperm quality		Murdica et al. (2019); Xie et al. (2022)
		O-EVs have been shown to increase the survival rate of fresh and cryopreserved sperm	O-EV	Alcântara-Neto et al. (2020a)
		Polyspermic fertilization was prevented by porcine O-EVs		Alcântara-Neto et al. (2020b)
		Cat O-EVs contain proteins involved in energy metabolism, reproductive function, and membrane modification, which can bind to sperm and improve sperm motility and fertility		Ferraz et al. (2019)
		PMCA4 can be transported to sperm by mouse O-EVs, V-EVs, and I-EVs, which promote calcium efflux and induce sperm capacitation. In the absence of PMCA4 in sperm, O-EVs could compensate by transporting PMCA1		Al-Dossary et al. (2013), Okunade et al. (2004)
		Mouse O-EVs can deliver miRNAs required for fertilization to sperm, such as miRNA-34c-5p, which is required for the first cleavage		Fereshteh et al. (2018)
		SPAM1 can be delivered to the sperm membrane by mouse I-EVs, promoting sperm fertilization	I-EV	Griffiths et al. (2008a)
		EM-EVs in the proliferative phase would be more suitable for promoting sperm fertilization, possibly because EM-EVs produced in the proliferative phase promote protein tyrosine phosphorylation in sperm	EM-EV	Murdica et al. (2020)
		Murine V-EVs may protect sperm from premature capacitation	V-EV	Fereshteh et al. (2019)
		EVs produced by mouse egg cells has been shown to promote sperm-egg fusion	Egg-EV	Miyado et al. (2008)
		FF-EVs can improve sperm motility and fertilization ability	FF-EV	Sysoeva et al. (2021)
		Bovine FF-EVs can induce sperm capacitation and acrosome reaction, but this ability is lost after trypsin treatment		Hasan et al. (2021)
Embryonic development		Embryo implantation in cattle changes the composition of O-EVs	O-EV	Mazzarella et al. (2021)
		Porcine O-EVs can increase ICM/TE, improve embryo adhesion, and increase the expression of genes involved in embryo implantation and reprogramming		Fang et al. (2022a)
		Melatonin found in rabbit O-EVs can increase blastocyst rate and hatching rate of embryos, decrease reactive oxygen species and 5-MC levels, increase ICM/TE, and reduce embryonic cell apoptosis		Qu et al. (2020)
		Mouse O-EVs can inhibit embryonic cell apoptosis, promote differentiation, and increase the birth rate after embryo implantation		Qu et al. (2019)
		Porcine O-EVs may improve the blastocyst formation rate and total cell number of parthenogenetic embryos by alleviating endoplasmic reticulum stress		Fu et al. (2022)
		To improve the quality and hatchability of embryos, bovine O-EVs can increase the influx of TCA cycle by up-regulating the expression of PDH and GLUD1 and down-regulating the expression of PDK4 and SIRT4		Sidrat et al. (2020)
		O-EVs can boost blastocyst formation and increase embryo survival time		Almiñana et al. (2017)
		Porcine O-EVs can increase blastocyst formation and hatching rates in parthenogenetic embryos, decrease reactive oxygen species, lipid content, and apoptosis in blastocysts, down-regulate BAX expression, and up-regulate BCL2, SOD1, SOX2, and other genes		Fang et al. (2022b)
		Bovine O-EVs have been shown to improve the survival rate of frozen embryos		Lopera-Vasquez et al. (2017)
		Bovine O-EVs can improve the re-expansion rate and hatching rate of frozen-thawed embryos, which may be related to O-EVs’ ability to restore trophectoderm integrity and improve junctional cell contact and fluid flux		Sidrat et al. (2022)
		After vitrification, freezing, or heating, bovine O-EVs improved embryo survival, lipid content, and total cell number		Leal et al. (2022)
		Pregnant mice’s uterine cavity contains more I-EVs and higher levels of miRNA-21 than non-pregnant mice’s uterine cavity. miRNA-21 may be involved in embryonic development	I-EV	Lv et al. (2018)
		Cattle with endometritis have I-EVs that differ from normal I-EVs and are harmful to embryonic development		Wang et al. (2019)
		EV secreted from the bovine blastocyst stage contains miRNA-378a-3p, which has been shown to increase the number of embryonic trophoblast cells and inner cells and improve hatching rate	EO-EV	Pavani et al. (2022)
		Mouse SP-EVs can improve blastocyst formation rate and ICM/TE index in sperm/oocyte mixed medium, as well as reduce embryonic cell apoptosis	SP-EV	Ma et al. (2022)
Embryo implantation		I-EVs are produced during pregnancy in pigs by both embryonic trophoblast cells and endometrial epithelial cells, but primarily by endometrial epithelial cells. I-EVs during the porcine estrus cycle or on the 9th day of pregnancy can promote embryonic trophoblast cell proliferation and migration, whereas I-EVs on the 12th or 15th day of pregnancy can inhibit embryonic trophoblast cell migration and proliferation	I-EV	Hu et al. (2022)
		When endometrial epithelial cells and trophoblast cells were cultured with I-EVs from successful pregnancy pigs, the expression of ITGA5, CXCL10, and CXCL11 was significantly up-regulated, while ID2 expression was down-regulated		Ahmad et al. (2018)
		I-EVs can be used to assess early embryo implantation		Li et al. (2021)
		Ovine EM-EVs can increase IFNT production and promote trophectoderm cell proliferation	EM-EV	Ruiz-González et al. (2015)
		EM-EVs have the ability to increase trophoblast cell adhesion and invasion		Evans et al. (2019)
		miRNA-100-5p in EM-EVs may promote embryonic trophoblast migration and invasion by activating FAK and JNK in embryos, as well as angiogenesis during embryo implantation		Tan et al. (2020a)
		Differential miRNAs in EM-EVs produced by progesterone treatment of endometrial epithelial cells primarily regulate PI3K/AKT, BMP, and small RNA post-transcriptional silencing		Burns et al. (2018)
		EM-EVs produced by endometrial epithelial cells co-cultured with estrogen and progesterone contained more LAMA5 than EVs produced by endometrial epithelial cells cultured with estrogen alone. EM-EVs produced by E2 and P intervention of endometrial epithelial cells can improve embryonic trophoblast cell adhesion and up-regulate the expression of FAK and FAK combined with TYR397		Greening et al. (2016)
		In patients with RIF, EM-EVs can reduce the hatching rate of the embryo and weaken the embryo’s invasion ability. This could be because EM-EVs inhibit trophoblast cell proliferation, migration, and invasion		Liu et al. (2020a)
		EM-EVs produced by inflamed endometrial epithelial cells can inhibit the migration of bovine embryonic trophoblast cells and suppress the expression of CDX2, ER, and NANOG genes involved in early embryonic development in bovine embryos		Wang et al. (2021a)
		In mice, EM-EVs can increase the total cell number, hatching rate, and implantation rate of embryos		Gurung et al. (2020)
				Kim et al. (2019)
		EM-EVs and plasma EVs have the potential to aid in the diagnosis of RIF patients		Azhari et al. (2022)
				Rajaratnam et al. (2021)
				Zeng et al. (2021)
		MiRNA-34c-5p in mouse EM-EVs can be used to determine the best time for embryo implantation		Tan et al. (2020b)
		Progesterone-treated EO-EVs derived from bovine embryonic trophoblast cells could increase the expression of ITGAV, ITGA3, and WNT7A in endometrial epithelial cells while decreasing the expression of MUC1		Su et al. (2022)
		EO-EVs from RSA patients can transfer miRNA-196a-5p into macrophages, induce macrophage M1 polarization *via* the NF-κB pathway, and secrete TNF-α to inhibit trophoblast development	EO-EV	Zhang et al. (2022)
		EO-EVs can assess the viability of embryo implantation		Capalbo et al. (2016)
		Trophoblast EVs can promote EMT and migration of endometrial epithelial cells, recruit monocytes in a dose-dependent manner, promote the secretion of IL-1β, G-CSF, TNF-α, and IL-6, and enhance the ability of angiogenesis to promote embryo implantation, which may be the role of miRNA-1290		Shi et al. (2021)
		CAPG and AKR1B1 are both expressed in bovine EO-EVs and contribute to EMT.		Nakamura et al. (2016)
		EVs produced by trophoblast cells were able to recruit monocytes and promote the secretion of IL-1β, G-CSF, TNF-α, and IL-6 in a dose-dependent manner		Atay et al. (2011)
		EVs secreted by embryonic trophoblast cells can recruit mesenchymal stem cells in the endometrium and peripheral blood, potentially weakening the embryo’s maternal immune rejection		Calle et al. (2021b)
		SP-EVs can promote decidualization of endometrial stromal cells *in vitro*, which may be due to the proteins they contain activating the IL-11 signaling pathway	SP-EV	George et al. (2020)
		Endometrial epithelial cells were treated with SP-EVs, which increased the expression of genes involved in immunity and inflammation while decreasing the expression of genes involved in steroid biosynthesis, metabolism, and T cell differentiation		Bai et al. (2018)
		SP-EVs can stimulate endometrial epithelial cell production of IL-6 and IL-8		Paktinat et al. (2019)
		Endometrial epithelial cells treated with SP-EVs induced macrophages to secrete IL-1α and IL-6, while IL-10 secretion was suppressed		Paktinat et al. (2021)
		In aged mice, the implantation rate of embryos formed by semen and ovum after fertilization is reduced, which may be due to the weakened inhibitory effect of SP-EVs on middle dendritic cells in the uterus		Wang et al. (2021b)
		The number of M1 macrophages increased in RSA patients’ decidua. EVs released by M1 macrophages can up-regulate CDH1 expression while inhibiting CDH2 and VIM expression to prevent trophoblast cell EMT, which may be caused by miRNA-146a-5p and miRNA-146b-5p contained in EVs, which down-regulate TRAF6 expression	Decidua-EV	Ding et al. (2021)
		EVs produced by decidual stromal cells can increase trophoblast cell invasion, which may be caused by increased expression of SMAD2, SMAD3, and CDH1		Liu et al. (2020b)

## 5 Extracellular vesicles’ clinical utility in assisted reproduction

Assisted reproductive technology is a medical aid that is widely used in the clinical treatment of infertility. It includes artificial insemination and *in vitro* fertilization - embryo transfer (IVF-ET). Although assisted reproductive technology can help many infertility patients, its success rate is still low and it is costly. As a result, it is critical to raise the level and quality of assisted reproductive technology. The EVs described above are critical messengers for embryo formation, development, and implantation. However, most current studies are limited to animals, and human applications have not been thoroughly researched.

### 5.1 Potential application of EVs in the prediction and culture of egg cells

The ultimate success rate of assisted reproductive technology will be determined by the quality of oocytes. Cumulus cells, oocytes, and granulosa cells secrete follicular fluid. Cell communication in follicular fluid is critical for oocyte development, fertilization, and development (Yang et al., 2020; Zakerkish et al., 2020). As a messenger carrying vital components, EVs can facilitate cell communication in follicular fluid. As a result, FF-EVs can react and affect oocyte quality. FF-EVs primarily reflect oocyte quality through its content (Rodrigues et al., 2019; Battaglia et al., 2020). For example, because the amount and type of miRNA and lipid carried by FF-EVs differ between high and low quality follicles (da Silveira et al., 2021; Gad et al., 2022; Zhang et al., 2021), miRNA expression in FF-EVs differ between oocytes with and without successful fertilization (Martinez et al., 2018). Thus, we were able to assess oocyte developmental potential and fertilization ability using FF-EVs. Second, we can use extracellular vesicles’ ability to convey information to intervene in oocyte culture using EVs at the site of oocyte development. FF-EVs have been shown in previous studies to promote the expansion of cumulus cells (Hung et al., 2015), stimulate the proliferation of granulosa cells (Hung et al., 2017), and improve the developmental ability of oocytes (Asaadi et al., 2021). However, O-EVs can accelerate oocyte maturation and promote cumulus cell proliferation (Lange-Consiglio et al., 2017; Wei et al., 2022). Interestingly, O-EVs were also able to change gene expression in oviduct epithelial cells, which may represent FF-EV’s ability to improve sperm survival and fertilization (Hasan et al., 2020).

### 5.2 Potential application of EVs in culture and prediction of spermatozoa

Male sperm, like oocytes, is essential in artificial insemination and *in vitro* fertilization-embryo transfer. As a result, prior to using assisted reproductive technology, sperm must be optimized. EVs can have a significant impact on sperm survival and fertilization as a messenger in seminal plasma, oocyte transfusion fluid, and uterine fluid (Griffiths et al., 2008b; Ronquist, 2012; Al-Dossary et al., 2015). SP-EVs can protect sperm from maternal immune damage, increase sperm antioxidant capacity (Skibinski et al., 1992; Saez et al., 1998; Tarazona et al., 2011), improve sperm motility and fertilization capacity (Yang et al., 2017; Wang H et al., 2022), and improve sperm motility after cryopreservation (Rowlison et al., 2021). Furthermore, SP-EVs have some diagnostic value. SP-EV’s contents can reflect male infertility sperm status; for example, two tsRNA TRF-Val-AAC-010 and TRF-pro-AGG-003 in seminal plasma can be used to diagnose non-obstructive azoospermia (Han et al., 2022). SP-EVs, as messengers communicating with sperm in seminal plasma, can indicate the status of the sperm. Therefore, we can determine the quality of sperm using SP-EVs, making later use of sperm easier (Murdica et al., 2019). SP-EVs can help us understand the pathogenesis as well as evaluate the quality of the sperm. SP-EVs contain more miRNA-21-5p in incompetent pig sperm, which can inhibit sperm capacitation and provide new ideas for clinical treatment (Xie et al., 2022). O-EVs have been shown to improve sperm motility, promote sperm fertilization, extend sperm survival time (Ferraz et al., 2019; Alcântara-Neto et al., 2020a; Murdica et al., 2020), and reduce the occurrence of multiple fertilization (Alcântara-Neto et al., 2020b). I-EVs promoted sperm capacitation and acrosome reaction, whereas V-EVs inhibited sperm capacitation and acrosome reaction (Fereshteh et al., 2019). We wondered if the superposition effect of EVs in multiple reproductive anatomical parts on sperm would play a positive role in artificial insemination because of the effect of EVs in different parts of the female reproductive system. More intriguingly, FF-EVs promoted cumulus complex expansion and may induce oocyte maturation, whereas SP-EVs promoted sperm motility and spermatogenesis (Hasan et al., 2021; Abumaghaid et al., 2022). When FF-EVs are used to culture spermatozoa from asthenospermia patients, it can increase sperm motility and forward motility rate, improving spermatozoa fertilization ability (Abumaghaid et al., 2022).

### 5.3 Potential application of EVs in embryo culture and prediction

The oviduct is the site of zygote birth as well as preembryonic development. Current research indicates that O-EVs can improve embryo blastocyst formation rate, embryo survival rate, embryo hatching rate, and post-embryo transfer birth rate (Almiñana et al., 2017; Qu et al., 2019; Fang et al., 2022b; de Alcântara-Neto et al., 2022). Furthermore, O-EVs can improve embryo development after cryopreservation and survival rate after vitrification (Lopera-Vasquez et al., 2017; Leal et al., 2022; Sidrat et al., 2022). More than just O-EVs may influence embryo development *in vivo*. Other research has shown that SP-EVs and FF-EVs can also promote the development of *in vitro* embryos (da Silveira et al., 2017; Asaadi et al., 2021; Ma et al., 2022). This could pave the way for improved embryo culture and quality in the future. Another worthwhile application is that EO-EVs can promote embryo development when used to culture embryos (Qu et al., 2017; Pavani et al., 2022). Because both artificial insemination and *in vitro* fertilization embryo transfer rely on normal embryo development in the uterine cavity, the influence of the intrauterine environment on embryo growth cannot be overlooked. As previously stated, I-EVs from endometritis can inhibit embryonic development and have different contents than normal healthy I-EVs (Wang et al., 2019). As a result, we anticipate that we will be able to use the contents of I-EVs to evaluate the timing of the use of assisted reproductive technology.

### 5.4 Application and predictive potential of EVs in embryo implantation

Embryo implantation is a critical step in the completion of a pregnancy, which is unquestionably difficult for elderly women and patients with thin endometrium. After embryo transfer, embryo implantation failure will increase the psychological and financial burden on infertility patients. Of course, embryo implantation failure cannot be attributed solely to maternal factors. Poor embryo quality, poor developmental potential, and chromosomal abnormalities can all result in embryo implantation failure. Before implantation, the embryo will communicate with the mother’s uterus *via* EVs. However, there is no communication between the embryo and the mother prior to IVF-embryo transfer. We are unable to demonstrate that increasing the amount of time the embryo spends communicating with the mother increases the rate of implantation. However, recent research has shown that EVs produced by endometrial epithelial cells during the secretory phase can improve trophoblast cell adhesion (Greening et al., 2016; Evans et al., 2019) and increase embryo hatching and implantation rates (Kim et al., 2019; Gurung et al., 2020). This is because progesterone can increase the amount of EM-EVs and change its composition (Burns et al., 2018). Furthermore, EO-EVs can improve endometrial receptivity and regulate maternal immunity, facilitating implantation (Atay et al., 2011; Su et al., 2022). Although the role of seminal plasma in IVF is frequently overlooked, numerous studies have found that SP-EVs can regulate the maternal uterine immune system and create favorable conditions for embryo implantation (Paktinat et al., 2019; Wang et al., 2021b; Paktinat et al., 2021).

We strive to improve embryo quality and endometrial receptivity during IVF embryo transfer to ensure a successful pregnancy. The number of trophoblast cells, the number of inner cells, and the classification of cyst lumen are currently the main methods for screening high-quality embryos by embryo morphology, but embryo implantation potential is still unknown. Relevant studies have discovered that EO-EVs can alter the expression of specific transcripts in endometrial epithelial cells, such as the down-regulation of ZNF81 expression, but inferior embryos do not (Es-Haghi et al., 2019). Furthermore, miRNA-20a and miRNA-30c levels in EO-EVs were significantly higher than in unimplanted embryos, and both had some predictive value for embryo implantation (Capalbo et al., 2016). MiRNAs found in EVs in plasma and serum have been shown to have diagnostic value for embryo implantation in recent years (Rajaratnam et al., 2021; Zeng et al., 2021; Azhari et al., 2022). I-EVs can also indicate whether the endometrium is ready for embryo implantation. MiRNA-34c-5p, for example, is up-regulated in I-EVs during implantation (Tan et al., 2020b). In addition, miRNA-362-3p expression in I-EVs were significantly higher in non-pregnant patients following controlled ovarian stimulation-embryo transfer than in pregnant patients (Li et al., 2021). It can be concluded that during embryo transfer, we can perform noninvasive screening of embryos based on the signature content of EVs, and it can also be used to help with endometrial status assessment.

### 5.5 Through EVs, stem cells can regenerate reproductive capacity

EVs can act as messengers for stem cells, allowing them to repair and regenerate. The ability of stem cells to regenerate is primarily used in three areas: the restoration of female ovarian reproductive function, the restoration of male testicular reproductive ability, and the repair of female endometrial damage (Deng et al., 2019; Hong et al., 2022). Stem cell action pathways include homing, self-differentiation, paracrine, and immune regulation (Naji et al., 2017). EVs play a critical role in each process. For example, EVs can recruit stem cells (Liang et al., 2020; Wang D et al., 2022) and transmit stem cell information (Qu et al., 2022). Cells in the lesion site will release EVs in addition to recruitment factors to recruit surrounding tissues or peripheral stem cells (Chen et al., 2022). Stem cells can either release EVs to play a distant role or return to the lesion site to self-differentiate, replace damaged cells, and release EVs or other therapeutic factors (Fu et al., 2021). EVs released by stem cells have been shown to restore ovarian function (Liu et al., 2020c; Wang et al., 2021c), repair endometrial cells (Yao et al., 2019; Xin et al., 2020), improve testicular function (Deng et al., 2019). Despite extensive research in regenerative medicine, the mechanisms of action and clinical applications of stem cells in reproductive medicine remain unknown. Because EVs play an important role in the transmission of information between cells, they can assist us in deciphering the secret of stem cells for the restoration of reproductive ability.

## 6 Conclusion

EVs are shown in this review to play an important role in the reproductive system. The follicle, prostate, embryo, fallopian tube, uterus, and vagina all produce messengers that transport information to the follicular fluid, semen, fallopian tube fluid, uterine fluid, and vaginal secretions. The information carried by EVs are not constant; it can change depending on whether the reproductive system is healthy or sick. This information transfer can carry information not only from the sperm to the sperm, but also from the oocyte fluid to the sperm. Although current research on EVs in reproductive medicine is primarily focused on animals, the findings are expected to provide a new direction for the advancement of assisted reproductive technology. However, there is no denying that EVs raise many questions about how they reproduce. For example, What is the mechanism by which EVs are generated from cells? In terms of information transmission, how does it relate to the extracellular environment? Which receptors do target cells use to recognize EVs from different ligands? Is there a relationship between the EVs produced by different ligands and the EVs? These issues require further investigation in order to gain a better understanding of the physiological processes of sperm and egg cell development, embryo formation and development, embryo implantation, and so on.
